# Determinants of Cognitive Development in the Early Life of Children in Bhaktapur, Nepal

**DOI:** 10.3389/fpsyg.2019.02739

**Published:** 2019-12-06

**Authors:** Suman Ranjitkar, Mari Hysing, Ingrid Kvestad, Merina Shrestha, Manjeswori Ulak, Jaya S. Shilpakar, Roshan Sintakala, Ram K. Chandyo, Laxman Shrestha, Tor A. Strand

**Affiliations:** ^1^Child Health Research Project, Department of Pediatrics, Tribhuvan University Teaching Hospital, Kathmandu, Nepal; ^2^Department of Psychosocial Science, Faculty of Psychology, University of Bergen, Bergen, Norway; ^3^Regional Center for Child and Youth Mental Health and Child Welfare, NORCE Norwegian Research Centre AS, Bergen, Norway; ^4^Department of Community Medicine, Kathmandu Medical College, Kathmandu, Nepal; ^5^Department of Research, Innlandet Hospital Trust, Lillehammer, Norway; ^6^Centre for International Health, University of Bergen, Bergen, Norway

**Keywords:** cognitive development, Bayley scales of infant and toddler development, biological factors, socioeconomic factors, environmental stimulation, manual stepwise procedure

## Abstract

**Background:**

Children in low and middle income countries may have many risk factors for poor cognitive development, and are accordingly at a high risk of not reaching their developmental potential. Determinants for cognitive development in early life can be found among biological and socioeconomic factors, as well as in stimulation and learning opportunities.

**Objective:**

The present study aimed to identify determinants of cognitive, language and motor development in 6–11 months old Nepalese infants.

**Methods:**

Six hundred infants with a length-for-age *z*-score <-1 were assessed with the Bayley Scales of Infant and Toddler development, 3rd edition (Bayley-III). Information on socioeconomic factors, child and maternal demographics, clinical and biological factors, and the home environment were collected. In a manual stepwise variable selection procedure, we examined the association between selected biological, socioeconomic and stimulation and learning opportunity variables and the Bayley-III cognitive, language and motor development subscale scores in multiple linear regression models.

**Results:**

The length-for-age *z*-scores was positively associated with the cognitive composite score [standardized beta (ß): 0.22, *p* < 0.001] and the motor composite score [(ß): 0.14, *p* = 0.001]. Children born with low birth weight (<2500 g) scored significantly lower on all subscale scores. Diarrheal history was associated with poor language composite scores, and females had higher language composite scores than boys [(ß): 0.11, *p* = 0.015]. Children who had been hospitalized during the first month of life had also lower cognitive and motor composite scores than those who had not been hospitalized. Parental reports of physical punishment and lack of spontaneous vocalization were associated with poor cognitive and language composite scores, respectively. The statistical models with the various subscale scores as dependent variables explained between 8 to 16 percent of the variability in the cognitive developmental outcomes.

**Conclusion:**

Our findings reveal important determinants for developmental scores in infancy, and underline the role of biological risk factors faced by marginalized children in low and middle income countries such as in Nepal.

## Introduction

Children in low and middle income countries (LMIC) are at risk of not developing according to their potential, and this represents a major public health problem (McDonald and Rennie, 2011). The South Asian and sub-Saharan African regions have multiple poverty related risks such as malnutrition, poor health and poor quality of stimulation and learning environment for many children (Grantham-McGregor et al., 2007). In these settings, identifying the predictors for early child development will help in initiating early intervention plans to prevent developmental delays (Persha et al., 2007). Known biological risk factors for poor cognitive function that are common in LMICs include short gestational duration (Gutbrod et al., 2000; Espel et al., 2014), low birth weight (Tong et al., 2006; Gill et al., 2013; Donald et al., 2019; Sania et al., 2019; Upadhyay et al., 2019), anemia (Sungthong et al., 2002) and stunting (Haile et al., 2016; Woldehanna et al., 2017). Poor nutrition, one of the causes of stunting (De Onis and Branca, 2016), has crucial impact on the growth and development of the brain and later cognitive functioning (Georgieff, 2007; Georgieff et al., 2018). Early childhood illnesses like diarrhea have also shown to predict development in high risk children (Niehaus et al., 2002; Lorntz et al., 2006; Kvestad et al., 2015). There is also evidence that longer duration of breastfeeding enhances cognitive and language development in infants (Lee et al., 2016).

Indicators of socioeconomic status including economic conditions (Duc, 2009; Ribe et al., 2018) and parental education (Roberts et al., 1999; Duc, 2009) has consistently been associated with cognitive functioning (Christensen et al., 2014; Ribe et al., 2018). Adequate responsive stimulation during the first years of life is also crucial for children to reach their developmental potential (Yousafzai et al., 2016; Nguyen et al., 2018).

The first 1,000 days, lasting from conception to the end of the second year of early childhood, is a particularly important period for cognitive development (Bellieni, 2016). During this period, minor impairments of brain because of biological and psychosocial factors can affect the structural and functional development of the brain (Walker et al., 2011).

The current study is conducted in a low and middle income setting with multiple risks factors that might affect child development. We assessed the development of the children and collected information of potential predictors for child development such as biological, socioeconomic, stimulation, and learning opportunities for the children. The main aim of this paper is to identify the determinants of cognitive, motor and language development assessed with the Bayley-III in these Nepalese infants at 6–11 months old.

## Materials and Methods

### Study Design, Setting, and Population

The children were participants in a doubled blinded clinical trial entitled “The effect of Vitamin B12 supplementation in Nepali Infants on Growth and Development” (Strand et al., 2017) (ClinicalTrials.gov: NCT02272842). The study site is the Bhaktapur municipality and surrounding areas of Bhaktapur district in Nepal. We included 600 children aged 6–11 months who were at increased risk of stunting [length for age *z*-score (LAZ)<-1SD], who plan to reside in the area for the next 12 months and whose parents consented to participate. Children with severe illness requiring hospitalization, severe malnutrition (weight-for-length *z*-score<-3SD) and with severe anemia (Hb<7 g/dL) were excluded from the study. Those with ongoing acute infections such as fever or infection that required medical treatment were temporarily excluded and enrolled after recovery.

### Procedure

Enrollment and baseline assessments including Bayley test were done from April 2015 to February 2017. The children were identified by field staff from immunization clinics or through door-to-door home visits, and enrolled when their length was confirmed by a supervisor or a physician at the field office. Enrollment procedures included collection of demographic information of the families, length and weight taking, blood sampling and developmental assessments at the same day. After enrollment, the date of the home visit for the home inventory assessment was scheduled with the mother within 1-week. Ethical clearances was obtained from the National Health and Research Council (NHRC; No. 233/2014) in Nepal and from the Regional Committee for Medical and Health Research Ethics (REC; No. 2014/1528) in Norway.

### Outcomes

#### Cognitive, Language and Motor Development

The cognitive, language and motor development at baseline were assessed using the Bayley-III (Bayley, 2006a) This is a comprehensive assessment tool of developmental functioning in infants and toddlers aged 1–42 months, takes 40 to 60 min to administer and includes three main subscales; cognitive, language (receptive and expressive communication) and motor (fine and gross motor). The Bayley-III represents the gold standard in developmental assessment of this age group and is widely used for research purposes worldwide. We have used the American norms from a representative American sample (Bayley, 2006b). The raw scores of each subscales were converted into scaled scores with a mean of 10 (SD: 3) and a range from 1 to 19 and again converted to the three composite scores with a mean of 100 and standard deviation of 15. The scales for the current study was initially adopted for a study in the same population for children 6 to 24 months (Murray-Kolb et al., 2014), and found to be reliable and feasible in children between 6–11 months in the same study setting (Ranjitkar et al., 2018).

### Determinants

#### Baseline Information

Baseline information was collected within a week from the date of enrollment. The information included family socioeconomic factors, child and maternal demographics, clinical and biological factors and the stimulation and learning opportunities in home environment. At enrollment, length and weight of the child and the mother along with head circumference of the child, was measured by well trained field staffs at the clinic following standard guidelines. Birth weight was recorded according to the parental report. Similarly, blood samples were collected from all the children. Details of the study procedure have been published elsewhere (Strand et al., 2017).

#### Home Environment

The Home Observation for Measurement of the Environment (HOME Inventory) (Caldwell and Bradley, 1984) is a structured assessment of the home environment that are indicators of stimulation and learning environment of the children. It is performed by a combination of direct observation and an interview with the mother or caregiver of the child at home by trained field staffs. We used selected items from a Bangladeshi adapted version of the Home Inventory that have been found to be a feasible tool in the same population in Nepal (Jones et al., 2017). In the current study, the structured assessment took approximately 20 min to complete with altogether 16 selected items from the original version of HOME Inventory including two items from the “Emotional and verbal responsivity” factor, two items from the ‘Avoidance of restriction and punishment’ factor, four items from the “Caregiver promotes child development” factor, two items from the “Organization of physical and temporal environment”, three items from the “Provision of appropriate play materials”, and three items from the “Opportunities for variety in daily stimulation” (Table 1).

**TABLE 1:** Variables assessed in multivariable regression models that measured the association with cognitive, language and motor composite scores of Bayley-III in 600 Nepalese children aged 6–11 months.

**Candidate variables**	**Continuous**	**Categorical**
**Biological**		
Length-for-age *z* score	*z* score	
Weight-for-age *z* score	*z* score	
Weight-for-length *z* score	*z* score	
Head circumference	cm	
Hemoglobin level	g/dL	
Low birth weight		<2500 gm: Yes or No
Total gestational week	Weeks	
Maternal height		<150 cm: Yes or No
Diarrhea		Yes or No
Hospitalization during 1st month		Yes or No
Gender		Male or Female
**Socioeconomic**		
Age of mother	Years	
Birth order		1st, 2nd or 3rd and higher
Type of family		Nuclear or joint
Number of family members under 5 years	Numbers	
Caste		4 categories
Father education		3 categories
Mother education		3 categories
Ownership of land		Yes or No
Ownership of house		Yes or No
Number of rooms used		≤ 2: Yes or No
Separate bedrooms		Yes or No
Ownership of own vehicle		Yes or No
Remittance from abroad		Yes or No
Indoor smoking		Yes or No
Regular ANC		Yes or No
Cigarette smoking by father		Yes or No
Alcohol consumption by father		Yes or No
Time spent on caring child by father	Hours	
**HOME Inventory**		
1. Caregiver spontaneously vocalizes to the child at least twice during the visit		Yes or No
2. Caregiver responds to child’s vocalization with a verbal response		Yes or No
3. Caregiver reports no instances of physical punishment during the past week		Yes or No
4. Caregiver does not scold or criticize the child during the visit		Yes or No
5. Caregiver tends to keep child within visual range and looks at the child quite often		Yes or No
6. Caregiver consciously encourages developmental advances		Yes or No
7. Caregiver structures the child‘s day		Yes or No
8. Caregiver believes the child‘s behavior can be changed or modified and is influenced by the parent’s behavior		Yes or No
9. When the primary caregiver is away, care is provided by one of three regular substitutes		Yes or No
10. Child is not cared for by another child (under 12 years of age)		Yes or No
11. There are some toys, tins, balls, dolls, slates, or material in the house that are appropriate play materials for the child		Yes or No
12. The child has a riding toy or some toy that provides gross motor stimulation		Yes or No
13. Caregiver provides toys or interesting activities for the child during the visit		Yes or No
14. There are some magazines, newspapers, or books visible in the house		Yes or No
15. The caregiver tells the child stories or nursery rhymes at least once a week		Yes or No
16. The caregiver sings to the child every day		Yes or No

### Training and Quality Control

Before the start of the study, psychologists responsible for assessing children in the study were trained and standardized in the use of the Bayley-III. A well experienced local psychologist served as “gold standard” during training and throughout the study period, and the study psychologists were required to achieve a high inter-rater agreement (ICC > 0.90) before testing study children. Seven percent of all sessions were scored by two examiners for quality assurances for Bayley with ICC’s ranging from 0.97–1.00 showing excellent inter-rater agreement. All the assessments were video recorded for further check ups when required, and for feedback from the supervising psychologists to the assessors. Any particular issues or challenges with the testing were discussed on weekly Skype-meetings with the supervising team from Norway (IK, MH) during the study period.

For the HOME Inventory, the field staff were trained and validated against a “gold standard” before the study start and they were required to achieve a good inter-rater agreement (ICC = 0.74). Seven percent of all assessments were double scored by the psychologist for quality assurance giving an ICC of 0.88.

Growth measurements and other major activities were also standardized before the study and 5% double scoring were carried out by supervisors during the study period.

### Statistical Analyses

Demographic characteristics are presented as numbers (N) and percentages (%), and by means and standard deviations (SD). We used multiple linear regression models to identify determinants of the Bayley-III scores. In these models, the composite scores of the cognitive, motor and language scales of the Bayley-III were used as dependent variables and the variables listed in Table 1 were considered for inclusion in the statistical models.

For the caste variable, we set Newar caste as the reference group, and categorized the remaining in three groups; the Brahmin/Chhetri, Tamang and “Others”. Hospitalizations during the first month of life and history of diarrheal episodes prior to enrolment were dichotomized as “Yes” or “No”. Alcohol consumption by father was dichotomized as “Yes” or “No”. We introduced all the items of the HOME Inventory separately as independent variables.

Variables for the statistical analyses were carefully selected in a manual, stepwise forward procedure as suggested by Hosmer and Lemeshow (Applied logistic regression, Second Edition). In short, the association between each candidate independent variable (Table 1) with the selected outcomes were initially assessed in unadjusted models. Variables that were significant at a *P* < 0.2 level were kept in multiple models while those that were non-significant in the initial crude assessment were re-introduced one at a time into these multiple models. Only variables that remained significant after this process were kept in the final models that are presented in the paper. This manual stepwise procedure was repeated for each composite score; cognitive, language and motor development. The statistical analyses were performed in STATA version 15 (STATA, College Station, TX, United States).

## Results

The mean age of the children was 8 months (SD: 1.7), 309 (51.5%) were male and 62 (10.4%) were born preterm. Approximately 28% of the study children had a history of diarrhea 1 month prior to enrollment and 9% were hospitalized mainly related to their low birth weight or because of jaundice during the first month of life. The mean age of the mothers was 27 (SD: 4) years. Of the mothers, 37% were illiterate or had an educational level up to grade 5. Nearly 70% of the children belonged to the Newar ethnic group. Approximately 52% of the families resided in their own house, and 47% of the families had their own land (Table 2). The mean composite scores of cognitive and motor subscales were close to the American norms while the language scale was 1 SD lower than the norms (Table 3).

**TABLE 2:** Baseline information of 600 participant Nepalese infants.

**Demographic features**	**Categories**	**Number/Prevalence**	**Mean/SD**
Mean age of the child in months (SD)			8 (1.7)
Male child		309 (51.5)	
Home delivery		23 (4.0)	
Cesarean section delivery		148 (29.6)	
Mean birth weight, gm^1^ (SD)			2787 (497)
Preterm birth		62 (10.4)	
Birth order	a. First	292 (48.7)	
	b. Second	229 (38.1)	
	c. Third and higher	78 (13.0)	
Low birth weight (<2500 gm)		119 (20.0)	
No diarrhea on month prior to enrollment		432 (72.4)	
Hospitalization during 1st month of life		54 (9.0)	
**Demographic characteristics**			
Mother’s age			27.0 (4.0)
Father’s age			30.0 (5.0)
Literacy of mother	a. Illiterate or up to grade 5	223 (37.2)	
	b. SLC and +2	261 (43.5)	
	c. Bachelor and higher	116 (19.3)	
Literacy of father	a. Illiterate or up to grade 5	212 (35.3)	
	b. SLC and +2	280 (46.7)	
	c. Bachelor and higher	108 (18.0)	
Occupation of mother	a. No working/Agriculture	374 (62.1)	
	b. Daily wage earner	90 (15.0)	
	c. Services/Self employed	136 (22.7)	
Occupation of father	a. No working/Agriculture	34 (5.6)	
	b. Daily wage earner	278 (46.4)	
	c. Services/Self employed	288 (47.9)	
Ethnic group	a. Newar	422 (70.3)	
	b. Brahmin/Chhetri	47 (7.8)	
	c. Tamang	96 (16.0)	
	d. Other	35 (5.8)	
**Socioeconomic status**			
Family staying in joint family		292 (48.7)	
Family residing in rented house		291 (48.5)	
Number of rooms in use by the household (≤2)		337 (56.2)	
Kitchen and bedroom same		298 (49.7)	
Family having own land		282 (47)	
Remittance from abroad		57 (9.5)	
Drinking water supply	a. Mineral water/Jar water	46 (7.6)	
	b. Tap water/Tanker water	533 (88.8)	
	c. Well, Hand pump or other	21 (3.6)	
Type of cooking fuel	a. Firewood/Kerosene	113 (18.8)	
	b. Gas	477 (79.5)	
	c. Electricity	10 (1.7)	
**Breastfeeding and nutritional status of infants**			
Exclusive breastfeeding for 3 months or more		280 (46.7)	
Exclusive breastfeeding for 6 months or more		64 (10.6)	
Underweight (weight-for-age *Z* score ≤2)		112 (18.7)	
Stunting (length-for-age *Z* score ≤2)		194 (32.4)	
Mean Hemoglobin, g/L (SD)			105.9 (9.3)
Anemia (hemoglobin<110 g/L)		385 (64.0)	

*^1^Among 579 infants from whom we have birth weight.*

**TABLE 3:** Bayley-III composite scores from children 6–11 months of age residing in Bhaktapur, Nepal.

**Scales**	**Mean**	**SD**	**Range**
Cognitive composite	97.7	10.2	60–130
Language composite	85.5	9.3	53–124
Motor composite	95.2	12.6	52–133

### Determinants of the Cognitive Composite Score

The cognitive composite score was positively associated with the length-for-age *z*-score. Children who were born with low birth weight (<2500 gm) had 5 points lower cognitive composite scores compared to children with birth weight in the normal range. Those who had been hospitalized during the first month of life had an average 4.7 points lower scores compared to those with no such history. A history of alcohol consumption in the father and reports of physical punishment during the past week were also predictors for the cognitive score (Table 4).

**TABLE 4:** Linear regression analysis to identify determinants of the Bayley-III cognitive composite score in Nepalese children 6–11 months.

**Cognitive composite**	**Coef.**	***P*-value**	**95% Confidence Interval**	**Beta**
Length-for-age *z* score	4.0	<0.001	2.4, 5.7	0.22
Low birth weight	–5.4	<0.001	−7.8, −2.9	–0.20
Hospitalization during 1st month of life	–4.7	0.003	−7.7, −1.6	–0.13
Alcohol consumption by father	–1.0	0.032	−2.0, −0.1	–0.09
Caregiver reports instances of physical punishment during the past week	–2.2	0.046	−4.3, −0.1	–0.09
Constant	109.5			

*R squared, 16.4%.*

### Determinants of the Language Composite Score

Female children had significantly higher language composite scores than male children. Low birth weight and head circumference were significantly associated with lower language scores. Those who had a history of diarrhea 1 month prior to enrollment had 2 points lower language composite scores than those who did not. Tamang and other castes had lower language scores than those belonging to the Newar caste. Children whose mother or caregiver did not show spontaneous vocalization to the child during the home observation had significantly lower scores than those who had mothers or caregivers that showed such stimulation (Table 5).

**TABLE 5:** Linear regression analysis to identify determinants of the Bayley-III language composite score in Nepalese children 6–11 months.

**Language composite**	**Coef.**	***P-*value**	**95% Confidence Interval**	**Beta**
Female	2.0	0.015	0.4, 3.6	0.11
Diarrhea history on month prior to enrollment	–2.2	0.012	−3.8, −0.5	–0.10
Caste				
Brahmin/Chhetri	0.2	0.892	−2.6, 3.0	0.01
Tamang	–4.5	<0.001	−6.6, −2.3	–0.17
Others	–4.0	0.016	−7.3, −0.8	–0.10
Head circumference	–0.6	0.044	−1.1, −0.1	–0.10
Low birth weight	–2.5	0.013	−4.5, −0.5	–0.11
No spontaneous vocalization to the child	–4.2	0.038	−8.2, −0.2	–0.08
Constant	111.6			

*R squared, 8.4%.*

### Determinants of the Motor Composite Score

The motor composite score was associated with the length-for-age *z*-score. Other predictors for the motor composite scores were being born with low birth weight, hospitalization during the first month of life and family ownership of house (Table 6).

**TABLE 6:** Linear regression analysis to identify determinants of the Bayley-III motor composite score in Nepalese children 6–11 months.

**Motor composite**	**Coef.**	***P*-value**	**95% Confidence interval**	**Beta**
Length-for-age *z*-score	3.0	0.001	1.2, 4.8	0.14
Hospitalization during 1st month of life	–6.5	<0.001	−9.9, −3.1	–0.15
Low birth weight	–4.1	0.002	−6.7, −1.4	–0.13
Ownership of house	–3.1	0.002	−5.0, −1.1	–0.12
Constant	106.6			

*R squared, 9.5%.*

## Discussion

In a high risk sample of young Nepalese children, biological, socioeconomic and home environment factors were associated with cognitive, language and motor development. The assessed risk factors are common in most LMIC. Approximately one third of our participating children were stunted (length-for-age *z*-score **≤**2) and approximately 10 percent were born preterm. Length-for-age *z*-score and low birth weight were the strongest predictors of the cognitive subscale. For the motor subscale, hospitalizations at 1 month of life and length-for-age *z*-score were the strongest determinants. Newar caste was set as reference group among the castes and caste was the strongest determinant for the language subscale. The cognitive score was associated mainly with the biological factors including growth and hospitalization along with a slightly significant effect of alcohol consumption by the father and reported physical punishment during the past week, while the language domain was associated with both biological and language stimulation and the motor composite was associated with both biological and socioeconomic factors. All models explained 8 to 16 percent variability for the Bayley-III subscales.

### Biological Determinants of the Developmental Outcomes

Biological risk factors were consistently associated with all the assessed developmental domains. For instance linear growth was associated with both cognitive and motor development in line with studies that have shown that stunting is one of the main factors of poor child development (Sudfeld et al., 2015; Miller et al., 2016). Stunting is one of the most used indicators for denoting malnutrition in early childhood (Perumal et al., 2018) and there are several evidences of cognitive impairment because of malnutrition (Nyaradi et al., 2013). Our findings supports the frequent use of length-for-age as a proxy for neurodevelopment.

Low birth weight was significantly associated with all subscales of the Bayley-III, in line with the known risk of low birth weight for neurodevelopmental outcomes (Aarnoudse-Moens et al., 2009; Oudgenoeg-Paz et al., 2017). Our study children showed lower scores on language development with decrease in head circumference. Head growth is consistently associated with cognitive development in previous studies (Gale et al., 2004; Silva et al., 2006). For instance, head circumference was a strong predictor of the Bayley scores at all eight sites of Mal-Ed study in which Nepal was one of the site (Scharf et al., 2018), however, some studies contradict to the consistencies of results associated with head circumference and cognitive development (Martyn et al., 1996; Dupont et al., 2018). We expected head circumference to show a more overall association across domains in the current study, there was, however, a lack of such associations. The slight differences between studies may be due to differences in study design, sample sizes, age at testing as well as inclusion of other variables.

Twenty-eight percent of the study children has a diarrheal history 1 month prior to enrollment. Both hospitalization and diarrheal history was associated with the cognitive and motor subscales which is in line with previous study results in North India showing decreased neurodevelopmental scores in children with diarrheal history (Kvestad et al., 2015). There may be both direct and indirect pathways from these biological risks and the adverse development. For instance infants who are infected with enteric diseases such as diarrhea during the golden thousand days are affected in the absorptive function of a healthy intestinal tract that is critical for the optimal growth and development of the body and brain (Petri et al., 2008). The consequences of diarrhea and hospitalization may also be mediated by what has been refered to as “Functional isolation” (Lozoff et al., 1998). As a result from the biological conditions, infants may be characterized by irritable and apathetic behavior, and are at risk for receiving less quality responsive care from its caregiver (Kvestad et al., 2015). Thus, biological risks may indirectly limit the stimulation received from the physical and social environment.

Female children have significantly higher language scores than males in the present study. Others have described that female children had better language development than males, especially in communication gestures and vocabulary development (Eriksson et al., 2012). A meta analysis on parent child language interactions showed that mothers talk more to their daughters than their sons (Leaper et al., 1998). Thus, gender differences in communication between parents and children might explain this result.

### Socioeconomic Determinants of the Developmental Outcomes

In our results, ownership of house is a predictor for motor development. Houses can be a reflection of economic status of families in a Nepalese context (Subba et al., 2014), and hence, to own a house is one of the most important indicators of socioeconomic status in this setting.

Compared to children in the Newar ethnic group, children from the Tamang group had lower scores on language development. The low score on language development in the Tamang and other castes compared to the Newar can also be verified in relation to differences in the socioeconomic status between these groups. In the study area, the Tamang group are mainly migrated people from neighboring districts. The economic basis for this group is mainly agriculture and other labor work like work in carpet factories (Ghimire, 2014). A study of socioeconomic status of indigenous people in Nepal revealed that Newars have relatively better socioeconomic conditions than other indigenous group including Tamangs (Subba et al., 2014) which may explain the differences we see in language development in our study. The observed differences between the ethnic groups can also be due to variability in communication habits between the groups (Leonard et al., 2009).

The locality of our study setting is rich in cultural activities, and alcohol consumption is very common in this setting (Maharjan and Magar, 2017). In our analysis, father’s alcohol consumption is associated with lower scores on the cognitive subscale. Alcohol consumption in parents have been shown to be related to lower cognitive achievements in children (Bennett et al., 1988; Nordberg et al., 1994).

### Stimulation and Learning Opportunities as Determinants of the Developmental Outcomes

Of the 16 items from the HOME Inventory that were included in the analyses, only two were significantly related to the Bayley-III scores in these young Nepalese children. Children whose caregiver reported physical punishment during the past week had lower scores on the cognitive subscales. This is in line with studies conducted in the United States, where physical punishment such as spanking predicted lower cognitive scores (Straus and Paschall, 2009; MacKenzie et al., 2013). The children whose caregiver did not vocalize spontaneously to the child scored significantly lower on the language subscale. This may be understood in light of the findings in a previous study in the same setting, that showed lack of awareness amongst Nepalese mothers about the importance of interacting with their children (Shrestha et al., 2019). Our results thus confirms the importance of early parent-child communication for early language development especially in vocabulary development (Topping et al., 2013).

A wide range of stimulation and learning opportunities were included, and with two exceptions, physical punishment and caregiver vocalization, none of them were associated with cognitive development. It may be that in this high-risk group at this early age biological risk factors have the largest immediate impact on the childrens development.

### Strengths

The large sample of 600 children is one of the main strengths of this study. The Bayley-III with cultural adaptations have already been tested in the same population and found to be a promising tool in this setting (Murray-Kolb et al., 2014; Pendergast et al., 2018; Ranjitkar et al., 2018). The study was further strengthened by standardization practices before the assessments (Ranjitkar et al., 2018), and double scorings with the gold standard during the study period to maintain the quality of the data and prevent the examiners drift. The standardized and reliable measurement of predictors including stimulation and learning opportunities and LAZ is also a strength.

### Limitations

The sample is a high-risk sample that is part of a clinical trial, and thus, it is not a population-based sample, and care should be taken before generalizing to the population as a whole. A comparison with a typically developing group of Nepalese children would have given additional insight into predictors of development, but was beyond the scope of the present study. One of our inclusion criteria was a LAZ<-1, which reduces the variability and may potentially alter the association between LAZ scores and Bayley scores. We believe, however, that we captured the LAZ-range where there is a linear relation between LAZ and Bayley-scores. This assumption is supported by findings from a study in young children in India where there was a linear association between LAZ and Ages and Stages Questionnaire (ASQ) scores up to -1 LAZ but not beyond that (Kvestad et al., 2015). Birth weight of the children and diahreal episodes were recorded based on the parental reports.

## Conclusion

Although our result showed that both biological and social factors were associated with developmental scores of these children, our study underline the role of biological factors faced by marginalized children in low and middle income countries such as in Nepal. Early intervention programs should be encouraged for overall development of children in LMIC setting.

## Data Availability Statement

The datasets generated for this study are available on request to the corresponding author.

## Ethics Statement

The studies involving human participants were reviewed and approved by the National Health and Research Council (NHRC; No. 233/2014) in Nepal and Regional Committee for Medical and Health Research Ethics (REC; No. 2014/1528) in Norway. Written informed consent to participate in this study was provided by the participants’ legal guardian/next of kin.

## Author Contributions

TS, MH, IK, and RC designed the study. RC, MU, SR, JS, RS, MS, and LS conducted the research and were responsible for the field implementation and data collection. TS and SR analyzed the data and interpreted the results. SR, MH, IK, and TS had primary responsibility for the final content. All the authors read and approved the final version of the manuscript.

## Conflict of Interest

The authors declare that the research was conducted in the absence of any commercial or financial relationships that could be construed as a potential conflict of interest.
